# RecovGait: Occluded Parkinson’s Disease Gait Reconstruction Using Unscented Tracking with Gated Initialization Technique

**DOI:** 10.3390/s25227100

**Published:** 2025-11-20

**Authors:** Chiau Wen Yeong, Tee Connie, Thian Song Ong, Nor Izzati Saedon, Ahmad Al-Khatib, Mahmoud Farfoura

**Affiliations:** 1 Faculty of Information Science and Technology, Multimedia University Melaka, Melaka 75450, Malaysia; yeong.chiau.wen@student.mmu.edu.my (C.W.Y.); tsong@mmu.edu.my (T.S.O.); 2 Centre for Image and Vision Computing, COE for Artificial Intelligence, Multimedia University, Melaka 75450, Malaysia; 3 Centre for Advanced Analytics, COE for Artificial Intelligence, Multimedia University, Melaka 75450, Malaysia; 4 Department of Medicine, Faculty of Medicine, Universiti Malaya, Kuala Lumpur 50603, Malaysia; izzati@um.edu.my; 5 Faculty of Science and Information Technology, Al-Zaytoonah University of Jordan, Amman 11733, Jordan; ahmad.alkhatib@zuj.edu.jo (A.A.-K.); m.farfoura@zuj.edu.jo (M.F.)

**Keywords:** Parkinson’s disease, computer vision, occlusion, AlphaPose, unscented tracking, gated initialization

## Abstract

Parkinson’s disease is a neurodegenerative disorder disease that worsens over time and involves the deterioration of nerve cells in the brain. Gait analysis has emerged as a promising tool for early detection and monitoring of Parkinson’s disease. However, the accurate classification of Parkinsonian gait is often compromised by missing body keypoints, particularly in critical regions like the hip and legs that are important for motion analysis. In this study, we propose RecovGait, a novel method that combines a gated initialization technique with unscented tracking to recover missing human body keypoints. The gated initialization provides initial estimates, which are subsequently refined through unscented tracking to enhance reconstruction accuracy. Our findings show that missing keypoints in the hips and legs significantly affect the classification result, with accuracy dropping from 0.8043 to 0.5217 in these areas. By using the gated initialization with an unscented tracking method to recover these occluded keypoints, we achieve an MAPE value as low as 0.4082. This study highlights the impact of hip and leg keypoints on Parkinson’s disease gait classification and presents a robust solution for mitigating the challenges posed by occlusions in real-world scenarios.

## 1. Introduction

Parkinson’s disease (PD) is a progressive neurodegenerative disorder disease that involves the deterioration of nerve cells in the brain. The disease primarily affects the central nervous system, where this neuronal deterioration leads to movement problems such as tremors, rigidity, bradykinesia and postural instability [1,2,3,4]. Despite extensive research, there is currently no cure for PD. The medication can only control and manage the symptoms of the disease.

The diagnosis and monitoring of PD often involve costly clinical assessments and imaging techniques, which can be prohibitive for many patients. PD often impacts motor control, particularly gait, due to its effects on a part of the brain called basalganglia that controls and regulates body movement [5]. Individuals with PD typically take small and shuffling walking steps, and the walking strides are shorter than a healthy individual. A reduction in arm swing and freezing of gait, which is by the sudden inability to move the feet forward, are also common symptoms of PD. These gait disturbances have been extensively studied, and multiple investigations show that gait patterns can be used as reliable biomarkers to distinguish PD patients from healthy individuals accurately [6,7,8].

In previous research, there are two major ways for classification of PD using gait analysis, which are an invasive method and a non-invasive method. Invasive methods involve wearable sensors and pressure sensors that are attached to the patient’s body and foot [9,10,11,12,13]. These methods normally provide high accuracy and detailed motion data, but they often cause discomfort to the patients, can be expensive and are generally impractical for long-term monitoring.

On the other hand, non-invasive methods have been gaining attention in recent years due to their ease of use and potential for remote screening. The methods often use video recordings or vision-based analysis to capture gait patterns [14,15,16,17,18]. The advantage of non-invasive methods lies in their unobtrusiveness and low-cost. This advantage allows for broader accessibility and patient compliance. Furthermore, the advancement of computer vision and deep learning techniques can extract meaningful features from videos, which become increasingly feasible for detecting PD patients gait abnormalities.

**Figure 1:**
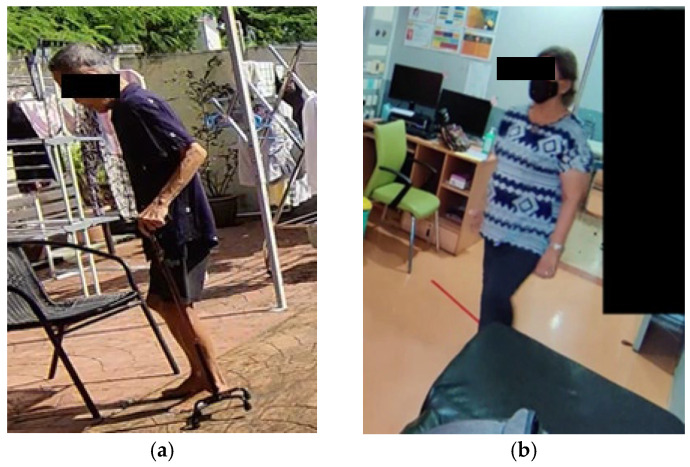
Examples of occlusions in real life. (**a**) The lower body of the subject is occluded by the walking stick and right leg is occluded by the left leg. (**b**) The lower part of the subject is being occluded by the furniture.

In computer vision, occlusion often refers to situations where parts of an object or person are partially or fully blocked by other objects or obscured due to a camera angle (refer Figure 1). Accurate detection of human body joints is critical for gait and posture analysis in PD screening. Models such as AlphaPose or OpenPose rely on the visibility of joint keypoints to extract meaningful motion patterns. However, occlusion often causes missing or incorrect keypoints, which can significantly degrade classification performance. These regions are often occluded because of self-occlusion during walking movements or blocking by other body parts or objects in the scene. In addition, motion blur can further obscure the visibility of joints in these areas. As a result, pose estimation algorithms may fail to accurately detect these keypoints, leading to incomplete skeleton keypoints representation and effect PD classification performance.

The aim of this study is to develop a low-cost method to recover missing keypoints caused by occlusion in full-body movement analysis for PD screening, leveraging computer vision and machine learning technique. While many prior studies focused primarily on gait-related features, this work extends the investigation to examine how occlusion in various body regions affect PD classification. Despite promising results from previous studies on vision-based methods, occlusion remains a major limitation. Environmental factors, such as walking aids or furniture, can temporarily hide body parts, leading to inaccurate PD classification results. Figure 1 shows some examples of real-life occlusions, where the patients are occluded by walking sticks or other objects, like furniture.

To address these challenges, this study proposes a method coined as RecovGait to recover missing keypoints using unscented tracking, enhanced by a gated initialization technique. The initialization step in unscented tracking is crucial and can significantly influence the accuracy of the predictions. It is improved by a lightweight gated initialization on providing robust initial estimate. The proposed method shows a promising result, achieving a MAPE score as low as 0.4082 in reconstructing missing leg keypoints, indicating highly accurate recovery.

## 2. Related Works

Gait and body movement abnormalities are the key indicators in recognizing the presence of PD. Changes in walking patterns, posture, and movement dynamics often serve as early and reliable signs of the disease. In recent years, various approaches have been deployed to classify PD patients using both wearable sensor data and vision-based techniques. This literature review explores the current methods in PD classification, focusing on video-based analysis, human pose estimation using computer vision and missing data reconstruction.

### 2.1. Parkinson Disease Detection and Classification

Recognizing PD through motion data has become an important research direction in healthcare. Recent studies leverage deep learning models such as Convolutional Neural Network (CNNs), Long Short-Term Memory (LSTMs) and Transformer to process time-series and spatial–temporal data from wearable sensors or video-based recognition.

Zeng et al. [17] presented a video-based approach using skeleton data and binary silhouette for quantitatively assessing gait impairments in PD using convolution network. The method utilized skeleton–silhouette fusion, where the 2D skeleton data were extracted using OpenPose and Long-Term Gait Energy Image (GEI). In addition, VGG network was deployed to extract silhouette feature vectors. Dual-branch CNN processed each modality separately before fusing the extracted features for final regression of impairment scores.

A study by Guayacán and Martínez [16] introduced a 3D convolutional gait for classifying Parkinson patients that used spatio-temporal patterns in classification. The dataset consisted of videos collected using a camera at 60 fps. There were two types of video input representations, namely raw video and optical flow. The videos were passed into 3D Convolutional Neural Network (3D CNN) to learn both spatial posture and temporal motion features relevant to Parkinsonian gait. The saliency maps were generated through backpropagation from the final convolutional layer to the input space. Interestingly, the visualization results consistently emphasize regions around the feet during the single-support phase.

A method proposed by Shin et al. [19] analyzed PD abnormal posture using the anterior flexion angle (AFA) and dropped head angle (DHA). The method applied a deep learning-based pose estimation algorithm on the data. AFA and DHA provided quantitative indicators to classify PD patients from healthy individuals. The author used OpenPose for tracking and segmenting the 2D position of ear, shoulder, hip and ankle. The high accuracy suggests that such pose-derived features could reliably be used in further classification tasks.

Yin et al. [20] assessed PD severity from videos using 3D CNN. They used transfer learning where the model was first pretrained on a non-medical dataset to learn robust visual and motion features. The network was then further finetuned on a video dataset. The addition of Temporal Self-Attention (TSA) improved focus on fine-grained temporal visual cues. Experimental results demonstrated the promises of using 3D data in PD analysis.

Although these studies show a promising result in detecting and classifying Parkinson’s disease using deep learning, several limitations remain. Most existing methods heavily rely on complete and high-quality motion data, but in real-world environments, occlusion, missing joints or truncated frames are common, which can significantly affect the results. On top of that, approaches that use CNNs and 3D CNNs primarily focus on spatial–temporal feature extraction but lack mechanisms on handling missing keypoints sequences, leading to unstable predictions when data are incomplete. Furthermore, some models also heavily depend on specific camera angles or manual feature definitions (e.g., AFA, DHA) could limit the generalization across datasets. Moreover, few studies explicitly address the reconstruction of missing keypoints as part of motion prediction.

### 2.2. Human Motion Estimation and Prediction

Human motion estimation and prediction are the key factors in recovering missing keypoints. Over the years, various approaches have been proposed, from traditional filtering methods to modern deep learning techniques to improve accuracy and robustness, especially when dealing with noisy data or incomplete observations.

A solution was presented by Yunus et al. [21] by using RNN-LSTM and Kalman Filter on human motion prediction. Feature extraction was performed by using OpenPose on extracting and generating 18 keypoints of human body pose. The extracted coordinates were converted to movement data. The movement data were further processed using RNN-LSTM and Kalman Filter to predict human motion. The accuracy was evaluated using Euclidean distance between two nodes from different frames.

Yang et al. [22] applied kinematic filtering based on UKF to the skeletal motion data captured by a Kinect v2 sensor. The primary objective was to enhance the accuracy of human motion estimation by combining the UKF’s predictive capabilities with noisy observational data from the sensor. In their approach, each joint’s position and velocity were modeled as part of a non-linear state–space system, where UKF uses to predict and update the joint states over time. As compared to the estimated value from UKF and ground truth, UKF had improved the accuracy of joint position tracking.

On the other hand, Bharathi et al. [23] employed attention-based LSTM pose estimation on a real-time human motion prediction. OpenPose library was used to extract the keypoints feature. Then, these keypoints were passed into LSTM network to capture the temporal dynamics of motion sequences. The LSTM network was fine-tuned and attention weights changed based on global features, where the changes were able to make the performance of the human motion prediction better.

Guo et al. [24] proposed a simple baseline for human motion predictions by using Multilayer Perceptron (MLP). The data used was a sequence of joint positions in 3D space. Some implementation details, such as encoding, normalization and data augmentation were conducted. A simple feedforward MLP was used by providing an input on a sequence of 3D joint positions and the model output for the predicted joint positions at future time steps. The model was evaluated using Mean per Joint Position Error (MPJPE). The evaluation was performed on the Human 3.6M, AMASS and 3DPW datasets. Experimental results have shown that their method outperformed existing methods.

Katircioglu et al. [25] introduced a dyadic human motion prediction using self-attention and pair-wise attention modules. The dataset was created by the authors, and called LindyHop600K, which consisted of videos and 3D human body poses of dancers performing. For each of the subjects, self-attention features and pair-wise attention were taken by the decoder as the input, and future 3D pose sequences as the output. The model used the past poses of two people and built a self-attention mechanism by adding a pair-wise module that links motions cues between both people. The module helped focus on relevant parts of the main subject’s movement based on the other person’s actions. The combined features went through GCN to predict the main subjects’ future poses. The proposed model achieved the lowest average MPJPE of 37.57 mm, outperforming all other variants across both short-term and long-term predictions.

While these studies have advanced human motion estimation and prediction, several limitations remain. Kalman Filter or UKF are highly dependent on accurate initial states and predefined motion models, which limit the adaptability when large gaps occur in the data. Conversely, deep learning models such as LSTM, MLP and attention-based networks can learn complex temporal dependencies but require complete datasets for training, and when there is a large portion of keypoints are missing, a more sophisticated network architecture is typically needed to maintain the result accuracy.

### 2.3. Missing Data Recostruction

Recovering missing keypoints due to occlusion is part of the broader task of missing data imputation. Therefore, the related works reviewed in this section primarily focus on studies involving data reconstruction and imputation techniques for human gait.

Gupta and Semwal [26] introduced different numerical methods on reconstructing occluded gait data under multi-person environments. The data were collected using Kinect sensor data which captured the full skeletal structure with 25 joints per subject. The data reconstruction focused on the lower body. For the occluded regions, interpolation and curve-fitting techniques were deployed to reconstruct the missing data. To evaluate the performance, error metrics such as MSE, RMSE, MAE and MAPE were used. Experimental results showed that Piecewise Cubic Hermite Interpolation outperformed others due to its non-linearity that was able to capture more complex and cyclic patterns of human gait.

Generative Adversarial Network (GAN) was widely used in data reconstruction [27,28]. Hasan et al. [27] implemented a GAN structure that included two main components, which are generator and discriminator. The generator used an encoder–decoder structure with 3D CNN layers. The encoder compressed the occluded silhouette into a compact latent vector, while the decoder reconstructed the missing parts. The discriminator learned to distinguish between complete silhouettes and reconstructed silhouettes using the generator.

On the other hand, Vernikos and Spyrou [28] had chosen LSTM as the discriminator to evaluate the reconstructed skeleton. Another method proposed by Kumar et al. [29] also detected occlusion before reconstruction. The authors first detected the occluded frames using a VGG16 network and preserved the non-occluded frames. Conditional variational autoencoder encoded each silhouette frame and used a key pose vector to create an effective latent representation. Bidirectional LSTM was used to capture forward and backward temporal dependencies to predict the occluded frames.

Jain et al. [30] introduced the fusion of forward and backward of LSTM. In forward LSTM, it took five non-occluded frames to predict the occluded frame. By leveraging both directions, the method effectively reduced errors compared to relying solely on past frames, which are often insufficient under heavy occlusions.

Singh et al. [31] presented a particle swarm optimization (PSO)-based neural network with multilayer features. Their reconstruction process was evaluated under four occlusion levels, ranging from 10% to 40%. The proposed method outperformed Artificial Neural Network and Alternating Least Square evaluated using MSE and MAPE.

Although various methods have been explored for reconstructing missing data, several limitations remain. Classical numerical techniques such as interpolation and curve-fitting relied heavily on assumptions of smooth and continuous motion, which often failed to capture non-linear motion in human motion. Deep learning-based reconstruction methods, such as GANs, VAEs and LSTM architectures demonstrated improved flexibility but depended heavily on well-annotated datasets and required sophisticated network architecture when the recovery of a large portion of data is required.

### 2.4. Summary and Research Motivation

In summary, existing studies demonstrated PD detection and motion analysis through both sensor-based and vision-based approaches. Deep learning methods have shown strong performance in modeling spatial–temporal motion features, but it typically rely on complete data, which making them less effective in real-world environments where occlusion or truncated frames occur. Traditional tracking methods like Kalman Filter and UKF provide stable tracking, but are highly dependent on accurate initialization and predefined motion models, where, when the data irregularities increase, the adaptability is limited. Similarly, reconstruction models like LSTM-based approaches tend to consume high computational power to achieve good reconstruction accuracy.

To overcome the limitations, the study introduces gated initialization tracking framework to improve motion recovery under missing keypoints. The gated initialization provides more accurate estimate and ensuring stable tracking performance even under severe occlusion. By incorporating lightweight gating mechanism within the unscented tracking, the method effectively balances computational efficiency with reconstruction accuracy. By integrating these, the proposed method enhances the robustness of PD classification in incomplete and real-world visual conditions, and addresses the key limitations found in previous works.

## 3. Proposed Solution

This section presents the details for the proposed approach for PD classification, with a particular focus on how missing keypoints from different body parts affect the classification accuracy. To capture the temporal dependencies inherent in sequential motion data, a Long Short-Term Memory (LSTM) network is employed as the primary classifier.

**Figure 2:**
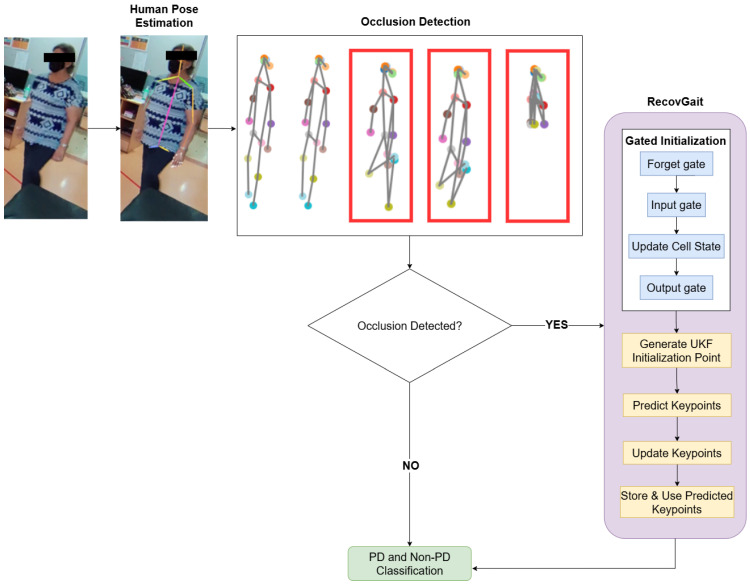
Overview of the proposed solution.

The proposed PD classification framework investigates the influence of occlusions by systematically analyses of how the absence of keypoints from different body regions affect the classification accuracy. To address the issue of missing keypoints, we propose an unscented tracking approach with gated initialization, known as RecovGait, for reconstructing occluded joints. Figure 2 illustrates an overview of the proposed method. Human pose estimation is first applied to extract the human skeleton keypoints from the video scene. Occlusion is detected based on structural inconsistencies among the extracted keypoints. Typical indicators of occlusion include unusual shrinking and collapsing toward nearby non-occluded joints. In addition, pose estimation provides a confidence score for each detected joint. Joints with a score below 0.5 are classified as occluded. Details of human pose estimation will be discussed in Section 3.2, occlusion detection will be discussed in Section 3.3 and RecovGait will be discussed in Section 3.4.

### 3.1. Dataset

**Figure 3:**
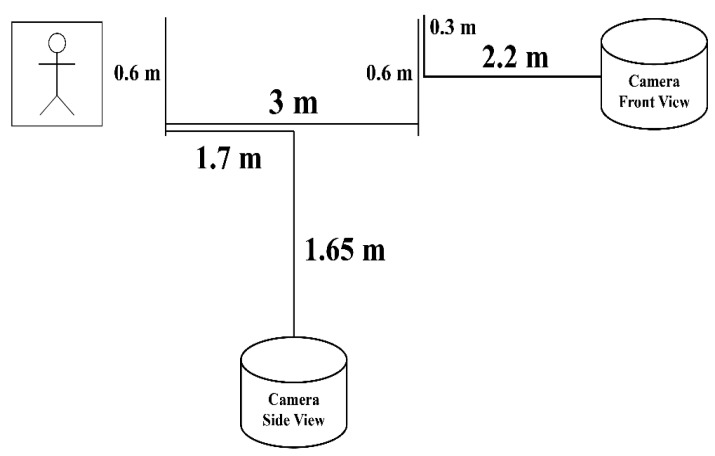
Data collection setup.

The dataset used in this study was self-collected following the Timed Up and Go (TUG) protocol. We contacted the PD patients for their consent to participate in the dataset collection. The data collection received ethical approval from the Multimedia University Research Ethics Committee (Approval Number: EA0422022). The setup of the experiment is presented in Figure 3. TUG test [32] is presented in the data collection process; TUG test is an assessment to determine a person’s mobility and fall risk, participants are required to sit, stand and walk. In the data collection process, participants are required to sit first, then stand and walk on a 3 m path back and forth to a sitting position, and two cameras are used to capture the walking pattern of the patients from the front and side of the patients.

The dataset is divided into two classes: PD and Healthy. The PD class consists of 26 patients while the Healthy class consists of 50 healthy individuals. To increase the dataset size and improve model generalization, data augmentation such as flipping, scaling and translation is further applied. After augmentation, the PD class has 104 samples, and the healthy class contains 200 samples. For training purposes, the dataset is split into 70% for training and 30% for testing.

### 3.2. Human Pose Estimation

The video data is processed using AlphaPose [33] to extract human skeletal keypoints. AlphaPose is a top-down multi-person pose estimator. It is the first open-source system that achieves 70 mAP on COCO dataset. There are two major steps in the pipeline, which are human detection and human pose estimation. It first detects the human bounding boxes using object detectors. Each of the detected bounding boxes are then cropped and resized. A pose estimation network is used to predict keypoints, and a re-identification network is applied to extract features for tracking. Symmetric Integral Regression is employed to localize the keypoints. To refine the results, non-maximum suppression is used to eliminate redundant pose detections. Finally, multi-stage identity matching integrates pose information, re-identification features, and bounding box data to produce a final tracking identity across frames.

**Figure 4:**
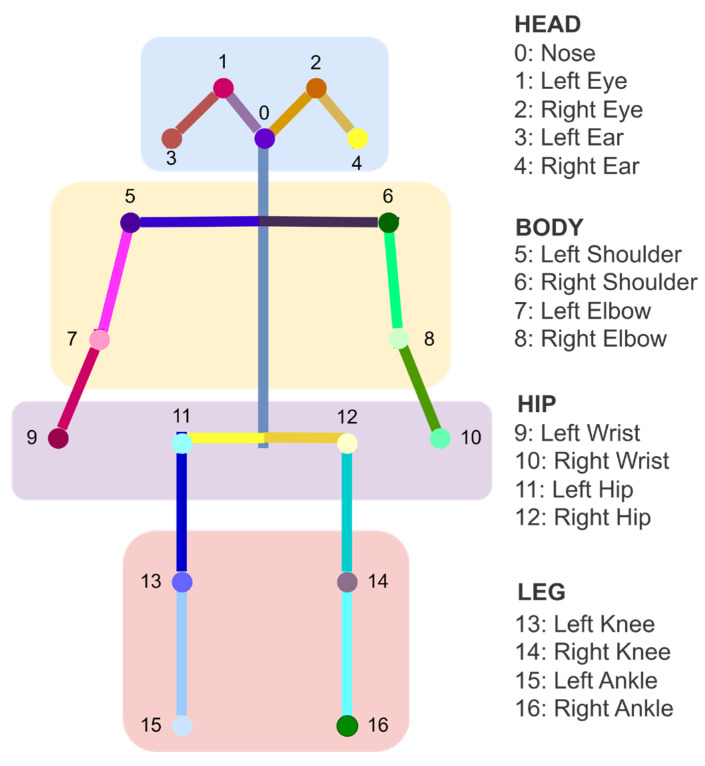
Illustration of human body estimation COCO17 keypoints.

In this research, the dataset utilizes the COCO 17 keypoint format, with the alignment keypoints shown in Figure 4. The 17 keypoints are divided into four main parts, where head includes keypoints 0 to keypoints 4, body includes keypoints 5 to keypoints 8, hip includes keypoints 9 to keypoints 12, and lastly, leg includes keypoints 13 to keypoints 16.

For the experiment, only 50 frames per video were extracted using AlphaPose. The extracted human keypoints from each frame are saved in a CSV file, organized sequentially from frame 1 till frame 50, with each frame containing keypoints 0 through 16.

### 3.3. Occlusion Detection

To detect occluded body keypoints, confidence scores from the pose estimation process are utilized. During human pose estimation, a confidence score is assigned to each keypoints i in every pose detection j. To obtain a robust estimate for each keypoint in the final merged pose, a score–weight merging strategy is employed. Let ${cluster\_scores}_{j,i}$ be the confidence score of keypoints i in pose detection  j, and ${masked\_scores}_{j,i}$ be the same score but masked to zero for detections that are too far from the reference pose. Here, k indexes all redundant poses in the cluster and i indexes the keypoints in the skeleton. The merged confidence score for keypoint i is computed as follows:

(1) $${merge\_score}_{i}=\sum_{j}\left({cluster\_scores}_{j,i}\times \frac{{masked\_scores}_{j,i}}{\sum_{k}{masked\_scores}_{k,i}}\right)$$

A keypoint i is classified as visible if

(2) $${merge\_score}_{i}>\tau$$

where τ is a predefined threshold. If the merged score falls below a predefined threshold τ, the corresponding keypoint i is labeled as occluded. This approach is robust to partial occlusion because the confidence scores of occluded joints typically decrease significantly, resulting in a low merged score.

### 3.4. RecovGait: Unscented Tracking with Gated Initialization

**Figure 5:**
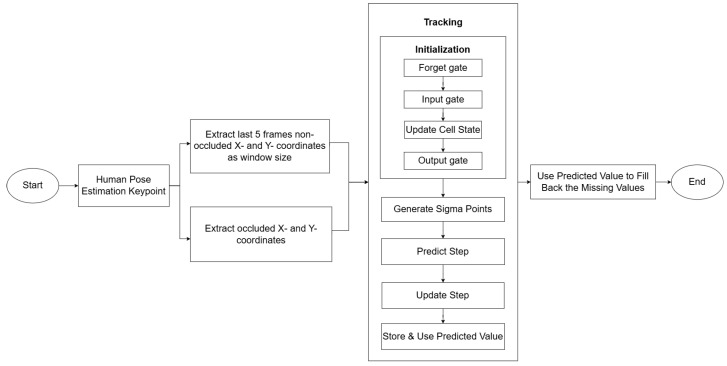
Flowchart of missing keypoints recovery using unscented tracking with gated initialization technique.

The core idea of the proposed RecovGait technique is to leverage the strength of unscented tracking and gated Recurrent Neural Network to improve the accuracy of missing keypoints recovery while maintaining low computational cost. Figure 5 shows the flowchart of the proposed recovery process. By integrating lightweight recurrent architecture with unscented tracking, the method achieves a balance between deep learning predictive power and unscented tracking computational efficiency. This makes the entire process not only accurate but also highly cost-effective.

#### 3.4.1. Initialization

There are several variations of the Kalman Filter [34]. One variant designed to handle non-linear data is the Unscented Kalman Filter (UKF), also referred to as unscented tracking [35]. Unscented tracking is a state estimation technique used for non-linear dynamic systems. Unlike the traditional Kalman Filter, which relies on linear approximations, unscented tracking uses unscented transform to capture the non-linearities in system dynamics. This feature makes it more suitable for estimating missing values in complex motion sequences, such as moving objects. It works by maintaining an estimate of the system state and updating it based on observed measurements. The key steps include a prediction step, sigma point generation, and updating the state.

Initialization is crucial to maintain accuracy of the tracking result. In this study, we propose a lightweight unscented tracking with gated initialization technique. The initial state estimation is defined as ${\hat{x}}_{0}$ and initial error covariance is defined as $P_{0}$. The initialization step is important as it influences the prediction accuracy, especially in a non-linear dynamics system,, such as human motion. Instead of relying on default values for initial state ${\hat{x}}_{0}\;$ and covariance $P_{0}$, the proposed gated initialization learns the patterns of gait from observed keypoints. The gated initialization technique allows the prediction to start from a state that is closer to the true value, particularly in sequences with missing keypoints.

A sliding window mechanism is used. A fixed-length window of five consecutive frames is used to generate input–output pairs. Specifically, each sequence of five frames serves as input to predict the subsequent frame, enabling the model to effectively capture local temporal dependencies. To evaluate the effect of network complexity on reconstruction accuracy, gated initialization networks with 30, 50, and 70 units are tested. The network architecture, as shown in Table 1, consists of a single gated initialization layer with ReLU activation 26 and a dense output layer with a linear activation function, to generate continuous numerical predictions. During training, the model is optimized using Adam optimizer with MSE as the loss function and learning rate is set at 0.001. Missing values are reconstructed iteratively using the trained model.

#### 3.4.2. Tracking

The initial parameters from initialization define the input for the prediction step, which is the state transition function. Once initialized, ${\hat{x}}_{0}$ and $P_{0}$ serve as the starting point of tracking and reconstruction process, then the subsequent tracking and predicting of missing keypoints are performed. State transition function models the temporal evolution of keypoints, allowing the system to reconstruct missing coordinates when visual observation is unavailable due to occlusion. The state transition function is defined as follows:

(3) $$x_{k}=f\left(x_{k}-1\right)+w_{k-1}$$

where $x_{k}$ is the system state vector at time step k, f(.) is the non-linear state transition function that is used to calculate predicted mean state $x_{k}$ and applying unscented transform, and $w_{k-1}$ is the process noise. In the prediction phase of tracking, state transition function is used to figure out the uncertainty in current state; it estimates how it will change over time. The measurement function allows the UKF to compare the actual measurement and predicted measurement; it is used directly after the prediction state, where the equation is given by

(4) $$z_{k}=h\left(x_{k}\right)+v_{k}$$

where $z_{k}$ is the measurement vector, h(.) is the non-linear measurement function, and $v_{k}$ is the measurement noise.

In sigma point generation, instead of relying on single Gaussian distribution, unscented tracking generates a set of sigma points to capture the mean and covariance of the prior state distribution. Equation (5) is the first sigma point, the mean of the distribution, and the best estimate of current state. Equation (6) is the next n sigma point, each created by adding a scaled portion of variance and mean. Equation (7) is the remaining n sigma point, created by subtracting the same vectors. Given a state vector $x_{k-1}\in R^{n}$ and its covariance $P_{k-1}$, the sigma points $X^{\left(i\right)}$ are computed as follows:

(5) $$X^{\left(0\right)}=x_{k-1}$$

(6) $$X^{\left(i\right)}=x_{k-1}+\sqrt{{\left((n+\lambda )P_{k-1}\right)}_{i},}\;\;\;i=1,\ldots ,n$$

(7) $$X^{\left(i+n\right)}=x_{k-1}-\sqrt{{\left((n+\lambda )P_{k-1}\right)}_{i},}\;\;\;i=1,\ldots ,n$$

where $P_{k-1}$ is the state covariance matrix is from the previous step, n is the dimension of state vector, and λ is a scaling parameter that controls the spread of sigma points.

After a set of sigma points are generated, they will propagate through the non-linear state transition function f(.) as shown in Equation (8). In this step, the state is predicted based on previous observations from the data using a non-linear motion model. Then, a new set of predicted sigma points will represent the possible position of the keypoints.

(8) $$X_{k|k-1}^{\left(i\right)}=f\left(X_{k-1}^{\left(i\right)}\right)$$

From these propagated sigma points, the predicted state mean and covariance are calculated by taking weighted averages over those points as follows:

(9) $${\hat{x}}_{k|k-1}=\sum_{i=0}^{2n}W_{i}^{\left(m\right)}X_{k|k-1}^{\left(i\right)}$$

(10) $$P_{k|k-1}=\sum_{i=0}^{2n}W_{i}^{\left(x\right)}(X_{k|k-1}^{\left(i\right)}-{\hat{x}}_{k|k-1}){\left(X_{k|k-1}^{\left(i\right)}-{\hat{x}}_{k|k-1}\right)}^{T}+Q$$

where $W_{i}^{\left(m\right)}$ are the weights for the mean and $W_{i}^{\left(x\right)}$ are the weights for the covariance.

Sigma points are mapped through the measurement function as shown in Equation (11); each predicted sigma points pass through measurement function h(.). The current set of sigma points now in the measurement space representing the predicted observations.

(11) $$z_{k}^{\left(i\right)}=h(X_{k|k-1}^{\left(i\right)})$$

The predicted observation mean is obtained as

(12) $${ẑ}_{k}=\sum_{i=0}^{2n}W_{i}^{\left(m\right)}X_{k}^{\left(i\right)}$$

Next, the innovation covariance and cross-covariance are computed as

(13) $$S_{k}=\sum_{i=0}^{2n}W_{i}^{\left(c\right)}{(z}_{k}^{\left(i\right)}-{ẑ}_{k}){{(z}_{k}^{\left(i\right)}-{ẑ}_{k})}^{T}+R$$

The cross-covariance between state and measurement sigma points are computed as

(14) $$P_{xz}=\sum_{i=0}^{2n}W_{i}^{\left(c\right)}{(X}_{k|k-1}^{\left(i\right)}-{\hat{x}}_{k|k-1}){{(z}_{k}^{\left(i\right)}-{ẑ}_{k})}^{T}$$

Using these, the Kalman gain is derived as

(15) $$K_{k}=P_{xz}S_{k}^{-1}$$

Finally, the state and covariance are updated with incoming measures.

(16) $$\hat{x}={\hat{x}}_{k|k-1}+K_{k}(z_{k}-{ẑ}_{k})$$

(17) $$P_{k}=P_{k|k-1}-K_{k}S_{k}K_{k}^{T}$$

where $K_{k}$ is the Kalman Gain, ${\hat{x}}_{k}$ is the updated state estimate after incorporating measurement and $P_{k}$ is the updated error covariance.

## 4. Experimental Result

### 4.1. Experiment Setup

The experiments were conducted on a laptop equipped with an Intel i5-9300H CPU 2.4 GHz and GPU NVDIA GeForce GTX 1650 Ti with Max-Q Design (Santa Clara, CA, USA). To investigate the impact of missing different body parts on PD detection, a lightweight gated initialization network was implemented. The detailed architecture of PD–Healthy Classification is shown in Table 2; it was structured as follows: an initial layer consisting of 128 units, followed by a dropout layer with a rate of 0.2 to prevent overfitting. A second layer with 64 units was then applied, followed by a dense layer with 32 units and another dropout layer at 0.2. The final output layer consists of a single neuron with a sigmoid activation function.

The model was compiled using the Adam optimizer with a learning rate between 0.00005 and 0.001 in different missing frames and different body parts, and binary cross-entropy as the loss function. An early stopping mechanism was applied to monitor the validation loss, with a patience value of 5 epochs to avoid overfitting and ensure efficient training. The model was trained for up to 50 epochs with a batch size of 32, using a training–validation split for evaluation. The model was saved at the best epoch that achieved the best validation performance.

### 4.2. Occlusion Simulation

Occlusion in human pose estimation often arises due to unfavorable camera angles or the presence of obstructing objects, which results in incomplete capture of the human body. To replicate such real-world conditions, an extended version of the original dataset was constructed by artificially removing selected body keypoints within a defined range of video frames, specifically between frame 10 and frame 40.

The extended dataset is stored in a CSV format, where each record corresponds to a single video consisting of 50 frames. For every frame, 34 keypoint values were extracted using AlphaPose, representing the x- and y-coordinates of 17 human body keypoints. Consequently, each record contained a total of 1700 data values (34 keypoints × 50 frames), representing the complete sequence of extracted coordinates for one subject.

**Figure 6:**
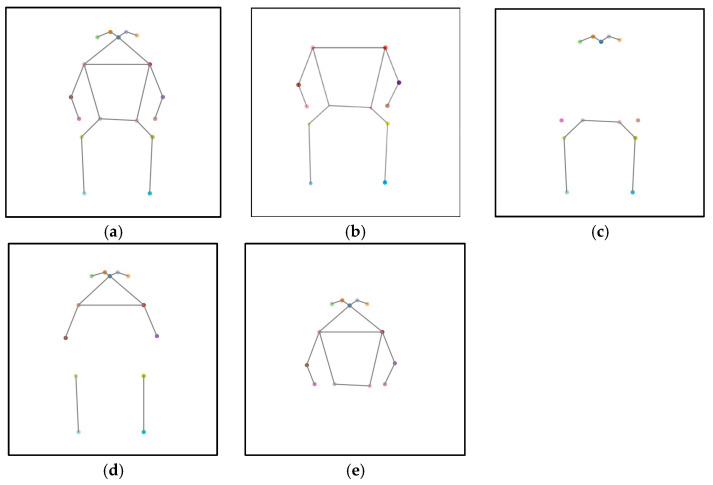
Illustration of missing keypoints in different body parts. (**a**) Illustration of full human body keypoints. (**b**) Illustration of missing head keypoints. (**c**) Illustration of missing body keypoints. (**d**) Illustration of missing hips keypoints. (**e**) Illustration of missing leg keypoints.

To further emulate real-world occlusions where specific body regions are blocked, the keypoints were grouped based on body parts: the head, upper body, hips, and legs. These groups of keypoints were selectively removed to simulate localized occlusion effects. Figure 6a illustrates the full-body keypoints of a patient, with each keypoint making up from the x- and y-coordinates. In the head region shown in Figure 6b, the occluded keypoints include the nose, left eye, right eye, left ear, and right ear. Each occluded keypoint was replaced with (x, y) = (0, 0) in the dataset. The occluded keypoints for the upper body shown in Figure 6c include the left shoulder, right shoulder, left elbow and right elbow. For the hip region shown in Figure 6d, the occluded keypoints include the left wrist, right wrist, left hip and right hip. Wrists were grouped under the hip region, since during walking, the hands (and wrists) typically align horizontally with the hips. The leg region shown in Figure 6e includes keypoints of the left and right knees, as well as the left and right ankles.

**Figure 7:**
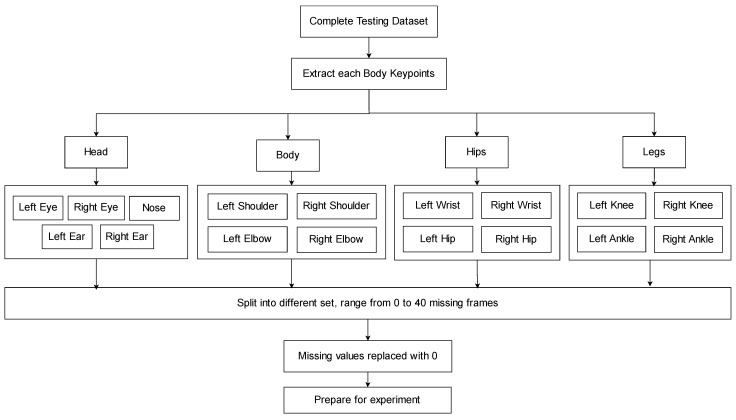
Flowchart of occlusion simulation in testing dataset.

Figure 7 illustrates the dataset preprocessing pipeline designed to simulate occlusion for testing modules, while the training modules utilize the complete dataset without missing values. The rationale for applying different preprocessing strategies to training and testing data is to assess how effectively the proposed technique can recover missing keypoints.

To simulate missing data caused by occlusions or sensor errors, specific keypoints are manually removed from the complete dataset. The processed dataset is then divided into five subsets: one with no missing values serving as the ground truth and four subsets with progressively missing frames, where the last 10, 20, 30, and 40 frames are removed, respectively. The removed values are replaced with zeros. This backward frame removal was used to simulate realistic challenges, as tracking methods usually rely on past frames to make predictions for the future state.

In addition, occlusions were simulated at the body-part level by independently removing grouped keypoints (head, upper body, hips, and legs). This enables analysis of which body region contributes most significantly to Parkinson’s disease classification. Once preprocessed as described, the dataset is ready for the next stage of experimentation.

### 4.3. Evaluation Metrics

To evaluate model performance, *Accuracy* was used as the primary metric, followed by *Precision*, *Recall* and *F1-score*, where the formula is as follows:

(18) $$Accuracy=\frac{True\;Positive+True\;Negative}{True\;Positive+True\;Negative+False\;Positive+False\;Negative}$$

(19) $$Precision=\frac{True\;Positive}{True\;Positive+False\;Positive}$$

(20) $$Recall=\frac{True\;Positive}{True\;Positive+False\;Negative}$$

(21) $$F1-Score=2\times \frac{Precision\times Recall}{Precision+Recall}$$

**Figure 8:**
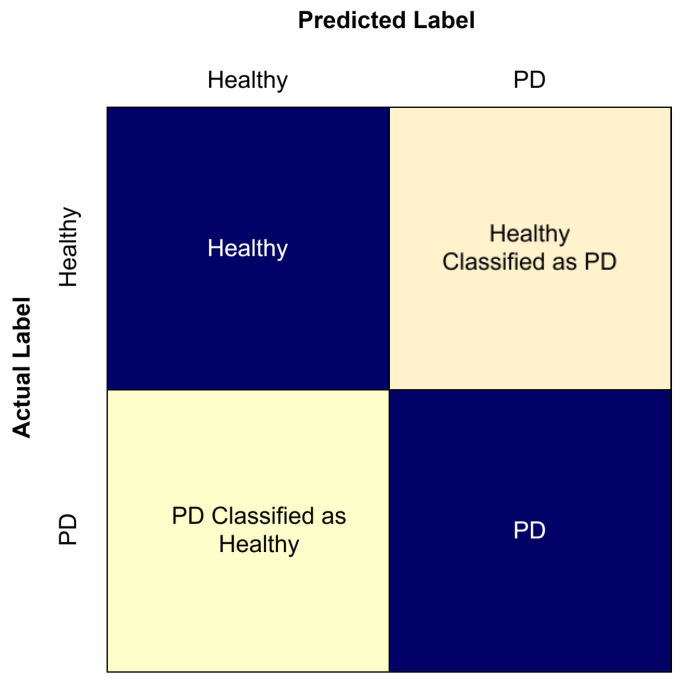
Confusion matrix.

A confusion matrix is used to provide a detailed overview of model’s classification performance by comparing predicted labels with the actual labels. Figure 8 shows a confusion matrix; each element in the matrix represents the number of samples belonging to a prediction–actual combination. In PD classification, the term true positive is defined as when the model correctly classifies PD, true negative is defined as when the model correctly classifies as healthy, false positive is defined as when the model incorrectly classifies healthy as PD, and false negative is defined as incorrectly classification of PD as healthy.

Several performance metrics are applied to assess the effectiveness of the proposed approach. The performance metrics used include Mean Absolute Error (*MAE*) [36], Mean Squared Error (*MSE*) [37], and Mean Absolute Percentage Error (*MAPE*) [38]. The formula for *MAE*, *MSE*, and *MAPE* are presented as follows:

(22) $$MAE=\frac{1}{n}\sum_{i=1}^{n}\left|y_{i}-{\hat{y}}_{i}\right|$$

(23) $$MSE=\frac{1}{n}\sum_{i=1}^{n}{\left(y_{i}-{\hat{y}}_{i}\right)}^{2}$$

(24) $$MAPE=\frac{100\%}{n}\sum_{i=1}^{n}\left|\frac{y_{i}-{\hat{y}}_{i}}{y_{i}}\right|$$

### 4.4. Evaluation of PD Classification with Occluded Parts

Table 3 presents the results of PD classification using LSTM under different occlusion scenarios. The complete dataset without any missing body parts or missing frames achieved the highest accuracy at 0.8913 and an F1-Score at 0.8387. From the overall comparison, leg and hip keypoints emerge as the most critical for accurate classification. In contrast, missing head and body keypoints still allow for moderate classification performance, which suggests that these regions are less sensitive to data loss.

The results also show that the effect of missing keypoints varies across different body regions. As shown in Table 3, an increasing number of missing frames consistently reduces classification accuracy across all groups. In the head region, the accuracy decreases from 0.8587 (10 missing frames) to 0.7609 (40 missing frames). The body region shows a decline from 0.8370 to 0.5870, while the hips region drops from 0.8478 to 0.5870. The leg region shows the most significant decline, from 0.8043 to 0.5217, as the number of missing frames increases from 10 to 40.

This downward trend, moving from head to legs, highlights the increasing importance of lower-body keypoints in PD classification. Since PD is strongly linked to gait and movement abnormalities, missing information in the leg region significantly undermines the model’s ability to distinguish PD patients from healthy controls. Thus, ensuring reliable recovery of leg and hip keypoints is essential for maintaining classification performance.

### 4.5. Missing Keypoints Recovery Ability

Table 4 shows the performance of the proposed method on an incomplete dataset with 40 missing frames and 70 LSTM hidden units across different body parts. The results show that the method remains effective even when a large portion of the data is unavailable. Among all body regions, the head exhibits the highest error rate, while the legs have the lowest error rate, indicating stronger recovery ability in the lower body.

**Figure 9:**
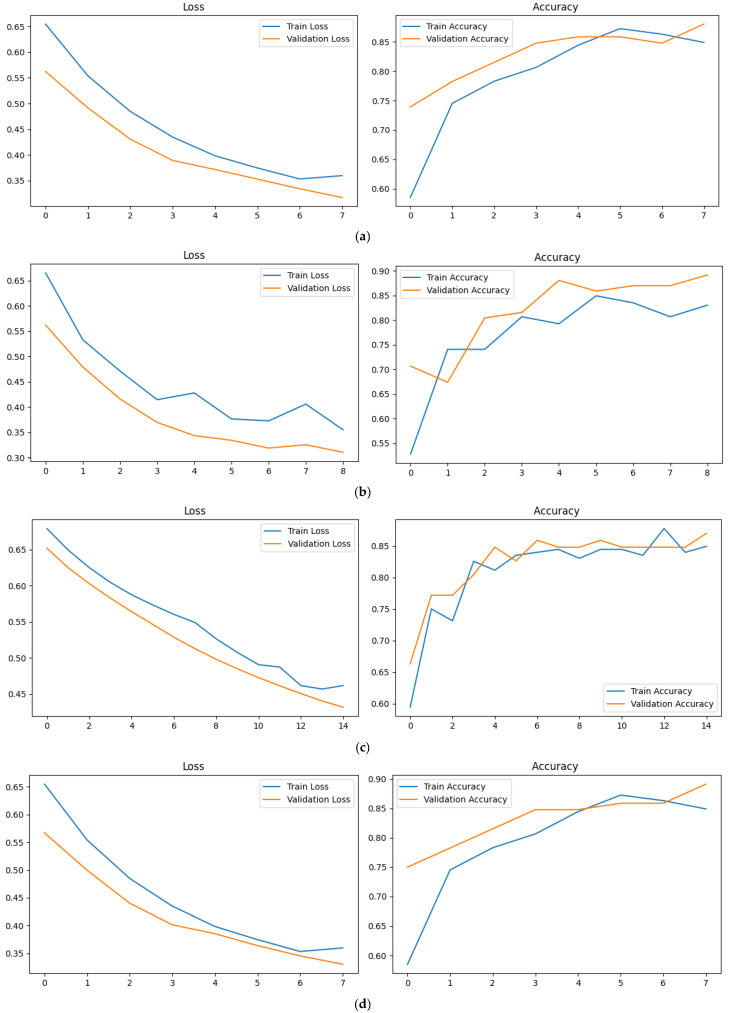
Loss curves and accuracy curves of PD Classification Training. (**a**) Head loss curves and accuracy curves. (**b**) Body loss curves and accuracy curves. (**c**) Hips loss curves and accuracy curves. (**d**) Leg loss curves and accuracy curves.

Table 5 shows the PD classification performance of the proposed method on an incomplete dataset with 40 missing frames and 70 LSTM hidden units across different body parts, and Figure 9 shows the loss curves and accuracy curves of PD classification. The results show that the proposed method is able to recover the classification performance after using the proposed method. The body, hips, and leg regions are able to recover a classification performance that is the same as the classification performance without occlusion.

Table 6 further evaluates the method under varying numbers of missing frames, ranging from 10, 20, 30 and 40 for each body part. The error increases progressively as the number of missing frames grows. The head region shows the most significant performance drop, highlighting its vulnerability to missing information. On the contrary, the legs and hips maintain relatively low errors even with 40 missing frames, demonstrating greater resilience to missing data.

Table 7 investigates the impact of different LSTM hidden unit sizes (30, 50, and 70) on recovery performance. Interestingly, the results reveal that the optimal performance does not always align with the largest LSTM size. For example, the legs achieve their lowest errors with 50 hidden units, while the hips perform best with 30 units. Across all configurations, the legs and hips consistently exhibit the highest predictability, whereas the head and body show greater sensitivity to model architecture. Collectively, these findings suggest that the proposed method effectively recovers missing keypoints, particularly in the hips and legs, which are the most crucial for PD-related movement analysis.

Table 8 shows the performance of the proposed method on an incomplete dataset with 40 missing frames and 50 LSTM hidden units under different levels of occlusion across different body parts. A performance decline was observed when more body parts were being occluded. When all the body keypoints are occluded, this represents the worst-case scenario of the occlusion.

### 4.6. Comparison with Other Methods

**Figure 10:**
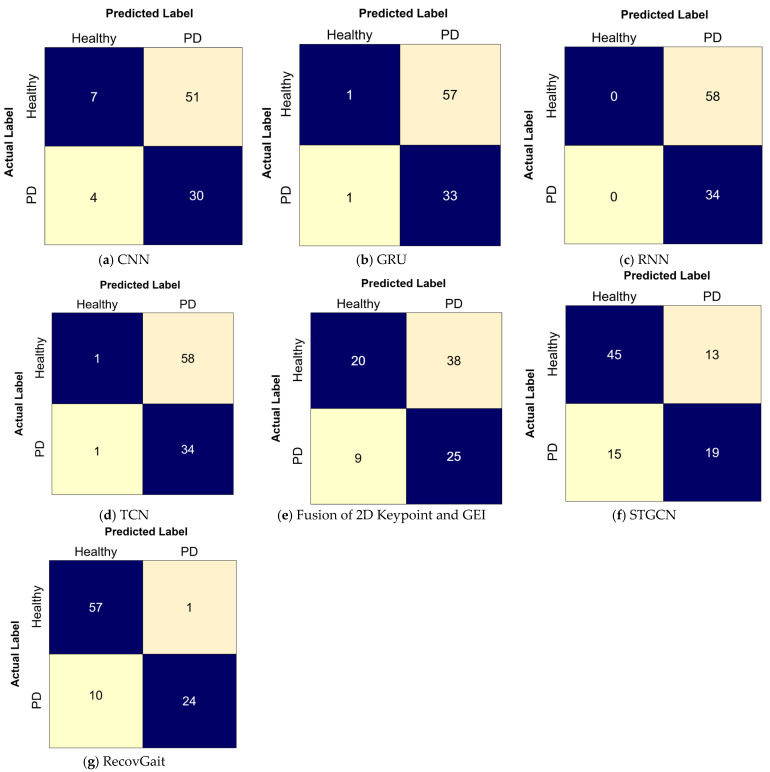
Confusion matrix of PD classification using RecovGait compared with other methods. (**a**) Confusion matrix of CNN. (**b**) Confusion matrix of GRU. (**c**) Confusion matrix of RNN. (**d**) Confusion matrix of TCN. (**e**) Confusion matrix of fusion of 2D Keypoint and GEI. (**f**) Confusion matrix of STGCN. (**g**) Confusion matrix of RecovGait.

Table 9 presents a comparison of four baseline models, namely Convolutional Neural Network (CNN), Gated Recurrent Unit (GRU), Recurrent Neural Network (RNN), Temporal Convolutional Network (TCN), Fusion of 2D Keypoint and GEI, and Spatio-Temporal Graph Convolutional Networks (STGCNs) against the proposed RecovGait framework in classifying PD, and Figure 10 shows the confusion matrix of all the methods. These models were selected because they capture key temporal and spatial dependencies in gait data and have been widely applied in recent studies on human activity recognition and PD detection. Unlike RecovGait, these baseline models rely solely on the classification stage and do not incorporate initialization or movement tracking features, making their performance more vulnerable to degradation when the input data contain missing keypoints. In contrast, RecovGait integrates gated initialization and unscented tracking, enabling a more stable performance under incomplete data conditions. All models were trained using the complete dataset and subsequently evaluated on datasets with missing keypoints.

The results clearly demonstrate that RecovGait achieves a significantly higher accuracy of 0.8804, whereas the best-performing baseline model attains only 0.6957 accuracy. This substantial performance gap highlights the effectiveness of RecovGait in overcoming the negative impact of missing data on classification accuracy. Overall, the comparison outlines the importance of integrating recovery mechanisms into the model pipeline, showing that RecovGait not only improves robustness but also provides a more reliable solution for real-world PD classification tasks where missing data is inevitable.

### 4.7. Visualization of the Proposed Method

**Figure 11:**
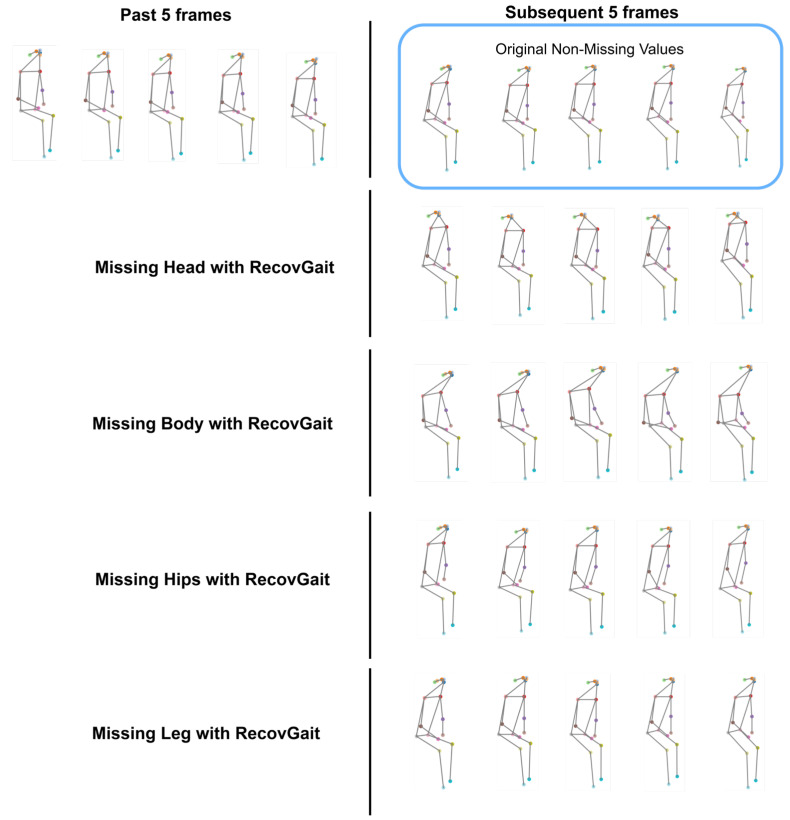
Visualization of missing keypoints recovery using RecovGait.

Figure 11 illustrates the visualization of the recovered missing keypoints using RecovGait. The first row shows the past five complete frames, with the subsequent five complete frames shown on the right side. The second row demonstrates recovery performance when five consecutive head keypoints are missing, and similar visualization applies for the other missing body parts using RecovGait.

From Figure 11, it can be observed that the leg region achieves the highest similarity to the original skeleton frames, consistent with the quantitative results in Table 4, where the legs recorded the lowest error values. The recovery accuracy follows a descending order: legs → hips → body → head. This visualization confirms the model’s ability to restore missing keypoints with high fidelity, particularly in the lower body, which is critical for gait-related tasks such as PD classification.

### 4.8. Ablation Study

To understand the contribution of each component in the proposed method, an ablation study is conducted as summarized in Table 10 and Table 11. The experiments were evaluated under four configurations: (i) without any recovery techniques, (ii) using only the unscented tracking method, (iii) using only the gated initialization model, and (iv) the proposed method combining unscented tracking with gated initialization. The evaluation was performed on sequences with the last 40 frames missing for each body part. For both the gated initialization model and the proposed method, a lightweight gated initialization model with 50 hidden units was employed to ensure fair comparison.

As shown in Table 10, the proposed method consistently outperforms the other configurations across all error metrics (MAE, MSE and MAPE). This demonstrates that the integration of unscented tracking with gated initialization enables the model to achieve accuracy levels comparable to more complex, higher-capacity architectures, while maintaining computational efficiency.

It is also observed that legs and hips exhibit lower recovery errors compared to head and body. The improvements for leg keypoints are particularly significant and visually more noticeable. This is likely due to the dynamic and periodic nature of leg movements in gait, which makes them inherently more predictable and well-suited for sequential modeling.

As shown in Table 11, the missing body parts keypoints severely degrade PD classification performance. The application of any technique resolves this issue, bringing the classification performance back to a robust level. This indicates that all methods are sufficiently recovering keypoints to enable correct high-level classification.

## 5. Discussion

The experiments conducted on LSTM-based PD classification and RecovGait for missing keypoint recovery provide several important insights:

- Several irregular performances can be observed in Table 3, particularly in head and body regions, where recall and F1-score increased despite a higher number of missing frames. These fluctuations were most frequent in head region, followed by body, hips and legs. This could indicate that head keypoints are less reliable and are the least uniquely informative for classifying PD and Healthy, so removing redundant head data could help the classifier. Legs carry stronger and more informative gait signals, so removing them is more likely to hurt the performance.
- Increasing the hidden units of gated initialization from 50 to 70 yields only marginal improvements across most body regions. This suggests that a lightweight module with 50 units is already sufficient to capture the underlying motion dynamics to enable lightweight yet effective recovery of missing keypoints.
- Both in classification and recovery experiments, missing keypoints from the legs and hips consistently lead to significant performance degradation. This shows the diagnostic importance of lower-body gait patterns in Parkinson’s disease, where motor impairments are often most evident in these regions.
- The results of PD classification in Table 11 can be observed to be unaffected by the recovery technique. This indicates that, to classify PD, a less precise keypoints recovery method was good enough. However, Table 10 shows that the proposed method was able to improve the overall data quality, and that higher-fidelity keypoints often lead to more reliable and noise-free feature vectors.

Although RecovGait was primarily developed to recover occluded gait keypoints in Parkinson’s disease analysis, the proposed framework demonstrates promising robustness and generalization characteristics. The gated initialization mechanism dynamically adjusts to varying occlusion patterns and confidence levels that enables reliable performance across different viewing conditions and keypoint detectors.

The framework is designed to be model-agnostic. It can integrate with any pose estimation model (e.g., OpenPose, BlazePose, MediaPipe) as it operates purely at the keypoint coordinate level. Future work will extend this analysis through cross-dataset validation and real-world video capture to further confirm generalization capability across diverse populations and camera environments.

## 6. Conclusions

In this study, we proposed a robust framework, RecovGait, which integrates unscented tracking with gated initialization mechanisms to recover missing human keypoints and enhance Parkinson’s disease (PD) classification from gait data. By simulating missing frames across different body regions, we evaluated both the impact of incomplete data on classification accuracy and the effectiveness of the recovery techniques. RecovGait exhibits particularly strong robustness under high missing-data conditions, most notably from the legs, which play a critical role in gait analysis for PD. The hybrid approach also proves to be highly computationally efficient, as it achieves a strong performance even with lightweight configurations. This makes RecovGait especially well-suited for deployment in resource-constrained environments where training and inference efficiency are essential. Moving forward, while this work relied on simulated missing data derived from benchmark datasets, an important avenue for future research is to validate RecovGait using real patient data captured from live camera systems in clinical or real-world settings. Such validation would better reflect practical challenges such as occlusions, motion blur, and hardware limitations, ultimately strengthening the clinical relevance and applicability of the proposed method.

While this study focuses on binary classification between Parkinson’s disease and healthy controls, the proposed RecovGait framework serves as a foundational step toward more complex tasks such as PD stage progression analysis. The accurate reconstruction of occluded lower limb keypoints ensures reliable gait features, which are important for distinguishing subtle motor variations across disease stages. Future work will extend RecovGait to multi-stage PD classification using clinically annotated datasets to enhance its clinical applicability and diagnostic value.

## Figures and Tables

**Table 1:** Detailed architecture of initialization.

Layer Type	Output Shape	Units	Activation Function
Gated Initialization Layer	(None, 50)	70	ReLU
Dense layer	(None, 1)	1	-

**Table 2:** Detailed architecture of PD and Healthy Classification.

Layer Type	Output Shape	Activation Function
LSTM	(None, 1, 128)	ReLU
Dropout Layer	(None, 1, 128)	-
LSTM	(None, 64)	ReLU
Dense	(None, 32)	ReLU
Dropout Layer	(None, 32)	-
Dense	(None, 1)	Sigmoid

**Table 3:** PD classification using LSTM.

Missing Body Parts	Number of Missing Frames	Accuracy	Precision	Recall	F1-Score
None	0 (Complete)	0.8913	0.9286	0.7647	0.8387
Head	10	0.8587	0.8889	0.7059	0.7869
	20	0.7826	0.6667	0.8235	0.7368
	30	0.8043	0.6739	0.9118	0.775
	40	0.7609	0.6250	0.8824	0.7317
Body	10	0.8370	0.7879	0.7647	0.7761
	20	0.7283	0.5918	0.8529	0.6988
	30	0.7609	0.6500	0.7647	0.7027
	40	0.5870	0.4697	0.9118	0.6200
Hips	10	0.8478	0.9167	0.6471	0.7586
	20	0.8043	0.7667	0.6765	0.7188
	30	0.6087	0.4848	0.9412	0.6400
	40	0.5870	0.4697	0.9118	0.6200
Legs	10	0.8043	0.6818	0.8824	0.7692
	20	0.7500	0.6486	0.7059	0.6761
	30	0.7391	0.7500	0.4412	0.5556
	40	0.5217	0.4638	0.9412	0.6214

**Table 4:** Missing keypoints recovery using the proposed method evaluation on body parts.

Missing Body Parts	MAE	MSE	MAPE
Head	7.8497	908.7418	1.9066
Body	5.0798	471.9854	1.4296
Hips	4.1824	317.5454	0.8116
Leg	2.3375	146.9356	0.4082

**Table 5:** PD classification using LSTM on missing keypoints recovery using proposed method evaluation on body parts.

Missing Body Parts	Accuracy	Precision	Recall	F1-Score
Head	0.8913	0.9000	0.7941	0.8438
Body	0.8804	0.8966	0.7647	0.8254
Hips	0.8913	0.9286	0.7647	0.8387
Leg	0.8913	0.9286	0.7647	0.8387

**Table 6:** Missing keypoints recovery using the proposed method evaluation on number of missing frames.

Missing Body Parts	Number of Missing Frames	MAE	MSE	MAPE
Head	10	3.6963	527.1320	0.8969
	20	5.7589	722.8728	1.4123
	30	6.8474	750.6274	1.6789
	40	7.8497	908.7418	1.9066
Body	10	2.4905	307.9000	0.6025
	20	3.8638	417.3950	0.9988
	30	4.8351	546.9760	1.2256
	40	5.0798	471.9854	1.4296
Hips	10	1.8295	182.4321	0.3533
	20	2.8317	250.7082	0.5623
	30	3.4392	289.3378	0.7007
	40	4.1824	317.5454	0.8116
Legs	10	0.6388	44.5666	0.1088
	20	1.0908	79.7515	0.1957
	30	1.9391	145.7927	0.3272
	40	2.3375	146.9356	0.4082

**Table 7:** Missing keypoints recovery using the proposed method evaluation on hidden units.

Missing Body Parts	Hidden Unit	MAE	MSE	MAPE
Head	30	8.1279	1198.0236	2.0085
	50	7.8789	898.5162	1.9147
	70	7.8497	908.7418	1.9066
Body	30	5.3704	556.6955	1.6043
	50	5.4319	612.5795	1.5647
	70	5.0798	471.9854	1.4296
Hips	30	4.0701	354.9734	0.8333
	50	4.9542	523.4678	0.8845
	70	4.1824	317.5454	0.8116
Legs	30	2.8210	261.0450	0.4687
	50	1.7369	122.5404	0.3194
	70	2.3375	146.9356	0.4082

**Table 8:** Different levels of occlusion from different body parts.

Missing Body Parts	MAE	MSE	MAPE
Leg	2.3375	146.9356	0.4082
Leg and Hips	8.1252	837.4347	1.5054
Body, Leg and Hips	13.4340	1388.0984	3.111
Head, Body, Leg and Hips	20.9866	2310.2282	4.9763

**Table 9:** PD Classification using RecovGait compared with other methods.

Method	Gated Initialization	Unscented-Based Tracking	Classification	Results
CNN [39]	N	N	Y	0.4022
GRU [18]	N	N	Y	0.3696
RNN [40]	N	N	Y	0.3913
TCN [41]	N	N	Y	0.3696
Fusion of 2D Keypoint and GEI [17]	N	N	Y	0.3891
STGCN [42]	N	N	Y	0.6957
RecovGait	Y	Y	Y	0.8804

**Table 10:** Ablation study in error metrics of missing keypoints recovery.

Technique	Missing Body Parts	MAE	MSE	MAPE
None	Head	148.7070	129,594.4808	23.5294
	Body	126.4514	113,363.3280	18.8235
	Hips	134.9398	121,589.3711	18.8235
	Legs	146.0150	133,479.4501	18.8235
Unscented Tracking	Head	9.9978	5273.7541	2.8017
	Body	7.8095	4288.3611	2.3881
	Hips	7.0212	4350.7643	1.6154
	Legs	5.0888	4504.3500	1.1482
Gated Initialization Model	Head	8.1903	939.1518	1.9956
	Body	5.7501	696.2457	1.5205
	Hips	6.0007	761.5744	1.0084
	Legs	3.2671	248.1791	0.6321
RecovGait	Head	7.8789	898.5162	1.9147
	Body	5.4319	612.5795	1.5647
	Hips	4.9542	523.4678	0.8845
	Legs	1.7369	122.5404	0.3194

**Table 11:** Ablation study in PD classification.

Technique	Missing Body Parts	Accuracy	Precision	Recall	F1-Score
None	Head	0.7609	0.6250	0.8824	0.7317
	Body	0.5870	0.4697	0.9118	0.6200
	Hips	0.5870	0.4697	0.9118	0.6200
	Legs	0.5217	0.4638	0.9412	0.6214
Unscented Tracking	Head	0.8913	0.9000	0.7941	0.8438
	Body	0.8804	0.8966	0.7647	0.8254
	Hips	0.8804	0.8966	0.7647	0.8254
	Legs	0.8913	0.9286	0.7647	0.8387
Gated Initialization Model	Head	0.8913	0.9000	0.7941	0.8438
	Body	0.8804	0.8966	0.7647	0.8254
	Hips	0.8913	0.9286	0.7647	0.8387
	Legs	0.8913	0.9286	0.7647	0.8387
RecovGait	Head	0.8913	0.9000	0.7941	0.8438
	Body	0.8804	0.8966	0.7647	0.8254
	Hips	0.8913	0.9286	0.7647	0.8387
	Legs	0.8913	0.9286	0.7647	0.7647

## Data Availability

The dataset used in this study is available at https://www.kaggle.com/datasets/teeconnie/mmu-visual-based-parkinsons-disease-dataset (accessed on 1 November 2025).
